# Decisions and disease: a mechanism for the evolution of cooperation

**DOI:** 10.1038/s41598-020-69546-2

**Published:** 2020-08-04

**Authors:** Carl-Joar Karlsson, Julie Rowlett

**Affiliations:** Department of Mathematical Sciences, Chalmers University of Technology and The University of Gothenburg, 41296 Gothenburg, Sweden

**Keywords:** Pure mathematics, Infectious diseases, Public health, Human behaviour

## Abstract

In numerous contexts, individuals may decide whether they take actions to mitigate the spread of disease, or not. Mitigating the spread of disease requires an individual to change their routine behaviours to benefit others, resulting in a ‘disease dilemma’ similar to the seminal prisoner’s dilemma. In the classical prisoner’s dilemma, evolutionary game dynamics predict that all individuals evolve to ‘defect.’ We have discovered that when the rate of cooperation within a population is directly linked to the rate of spread of the disease, cooperation evolves under certain conditions. For diseases which do not confer immunity to recovered individuals, if the time scale at which individuals receive accurate information regarding the disease is sufficiently rapid compared to the time scale at which the disease spreads, then cooperation emerges. Moreover, in the limit as mitigation measures become increasingly effective, the disease can be controlled; the number of infections tends to zero. It has been suggested that disease spreading models may also describe social and group dynamics, indicating that this mechanism for the evolution of cooperation may also apply in those contexts.

## Introduction

Decisions made by individuals affect the population, not the least in disease spreading. Several researchers have investigated the interplay between diseases and decisions by combining compartmental models with game theory^1–5^. Common considerations are dynamics on networks or lattices^5–18^ and well-mixed populations^19–28^. The former’s strength is that it captures the effect of population structures, while the latter’s strength is that it highlights the individuals’ perception of the payoff. We consider a society in which individuals choose to what extent they will exert preventive measures to mitigate the spread of an infectious disease. The population may range from having a social network structure to being well-mixed. Individuals have two choices: exert mitigating measures to prevent the spread of the disease, and do-not-mitigate, making no efforts to prevent the spread of the disease. Individuals do not necessarily maintain the same choice; they are free to change their behaviors based on their perception of cost versus benefit. The World Health Organisation^29^ and numerous other references including^30–32^ argue that it is reasonable to describe this situation with the Prisoner’s Dilemma (PD).

**Figure 1:**
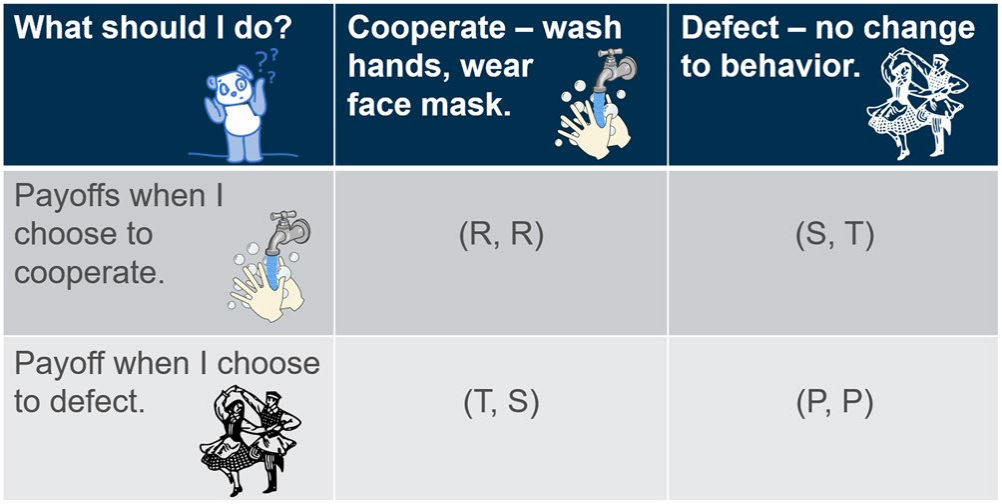
In the ‘disease dilemma’ people have the choice to cooperate, mitigating the spread of the disease, or defect, making no change to their regular behaviour. This is described by the non-cooperative game shown here in normal form. Image source and license: openclipart.org, CC01.0.

The payoffs may be interpreted as in Fig. 1. If both Alice and Bob defect, then they pay no cost but also receive no benefit, hence their payoffs are both $P=0$. Let us consider the particular example of cloth face masks. If Alice cooperates while Bob defects, then Alice pays the cost of buying the mask and enduring its discomforts, which is represented by $-B<0$. As demonstrated in^33^ Alice receives a small amount of protection from her mask, represented by $\epsilon > 0$, but the main benefit is reaped by everyone else. Consequently, if Bob does not wear a mask, he pays no cost but receives a benefit of $T> \epsilon > 0$. Alice’s total payoff is therefore $-B+\epsilon$. Since the benefit to Alice is relatively small, we assume further that $\epsilon < B$. If both Alice and Bob cooperate, then they both pay the cost $-B$, but they also receive the maximal protective benefit of $T+\epsilon$, and their total payoffs are thus $T-B+\epsilon$. Consequently, defining

$$\begin{aligned} C:= B - \epsilon , \end{aligned}$$

the payoffs satisfy

1 $$\begin{aligned} S= -C< P= 0<R< T = R+C. \end{aligned}$$

This particular representation of the Prisoner’s Dilemma is known as the Donor–Recipient game. The two-player game generalizes to a population-level model^34^ in which all individuals in the population choose whether or not to mitigate the spread of disease. In a well-mixed population, everyone interacts with each other, which is described by a fully connected graph. In reality, however, it is possible that certain individuals never interact, which can be described using a social network^34–37^. The presence of such a network modifies the payoffs (1), in such a way that if the social pressure is just right, cooperation may become more beneficial than defecting, an effect known as network reciprocity.

The payoffs (1) are modified by a quantity *N*(*k*), where *k* is the average node degree in the network, corresponding to the average number of social contacts each individual has. Making a standard set of simplifying assumptions as in^34–38^ allows us to incorporate the societal network structure and nonetheless obtain tractable expressions which are amenable to explicit analyses; see SI § “Network Reciprocity” for further details. The payoffs *R* and *P* remain as in (1), whereas the network structure now modifies the payoffs *S* and *T*

2 $$\begin{aligned} S = - C + N(k), \quad T = R + C - N(k), \quad N(k) := \frac{Rk -2C}{(k+1)(k-2)}, \quad k \in {\mathbb {N}} \setminus \{2 \}, \quad N(2) := R. \end{aligned}$$

The quantity *N*(*k*) increases with *R*, and tends to zero when $k \rightarrow \infty$. For intermediate values of *k*, this may be interpreted as social pressure, for example in a group of twenty persons all wearing masks, there is pressure to conform and also wear a mask. Consequently, *N*(*k*) increases the payoff value of *S* and decreases the payoff value of *T*. When $k \rightarrow \infty$, $N(k) \rightarrow 0$, and the model describes a well-mixed society. Although the model is still essentially based on pairwise interactions, dyadic games nonetheless are widely applicable to understanding societal level issues including but not limited to political crises^39^, vaccine compliance^40^, antibiotic resistance^41^, and cultural diversity^42^. Moreover, some argue that simple, transparent models from which insights are readily apparent may be of greater use than more complicated models^43^.

In the society-wide disease dilemma considered here, the unique equilibrium strategy is total defection. When this game is used to predict behaviours according to evolutionary game dynamics, the result is always defection^44^. Nonetheless, in many contexts which fit into a PD type game, cooperation may in fact be observed^45–51^. In the particular case of the PD, there have been numerous mechanisms proposed for the evolution of cooperation^38,52^. To our knowledge, it has been unknown—until now—whether cooperation emerges when the payoff is a trade-off between the PD and the effect on disease spreading through changes to the infection transmission rate.

## Methods

Infections like those from the common cold, flu, and many sexually transmitted diseases do not confer any long-lasting immunity, and individuals become susceptible once they recover from infection. These diseases are therefore described by the SIS compartmental model. Poletti et al.^53^ implemented a hybrid model in which human decisions affect the rate at which the disease spreads. They assigned two different rates of infection corresponding to individuals either changing their behaviour to mitigate the spread of the disease, or not doing so. We follow this approach by assigning the rates of infection for cooperators and defectors, $\beta _C < \beta _D$, respectively. In a population comprised entirely of cooperators, susceptible individuals become infected at the rate $\beta _C I(t)$. In a population comprised entirely of defectors, susceptible individuals become infected at the rate $\beta _D I(t)$. In both cases, *I*(*t*) represents the pool of infectious individuals. In a mixed population, where the proportion of cooperators is $x = x(t)$, and defectors is $1-x =1-x(t)$, susceptible individuals become infected at the rate $\beta (t) I(t)$, where

3 $$\begin{aligned} \beta (t)= (1-x(t))\beta _D+x(t)\beta _C. \end{aligned}$$

Since the portion of cooperators and defectors changes over time, the rate of infection, $\beta = \beta (t)$, is also a dynamical parameter, changing over time. The SIS-PD replicator equations with these considerations read

$$\begin{aligned} \frac{dI}{dt}&= \left[ (1-x(t))\beta _D + x(t)\beta _C \right] I(t)(1-I(t)) -\gamma I(t), \\ \frac{dx}{dt}&= x(t)(1-x(t))\left[ (\beta _D-\beta _C)I(t) -\left( [T-R]x(t)+[P-S](1-x(t))\right) \right] . \end{aligned}$$

Above, the quantities on the left side are differentiated with respect to $t=$ time. The rate at which infected individuals become susceptible again is $\gamma$. If *D* is the average duration of the infection, then $\gamma = 1/D$. We note that $1-I(t)$ is the portion of the population which is susceptible to infection, since in this model there is no immunity.

We would like to allow susceptible individuals to change their behavior spontaneously, corresponding to cost and benefit considerations. Choosing to mitigate, corresponding to the transmission rate $\beta _C$ incurs a cost, but reduces the risk of infection. Choosing not to mitigate increases the transmission rate of the population but does not incur any cost. The choice individuals make depends on the current state of the epidemic. It is important to note that the choice of behavior need not occur at the same time scale as the epidemic. The decision whether or not to take mitigation measures is based on the information to which one has access, via email, phone, internet, and media. On the other hand, epidemic transmission can occur only through interpersonal contact.

To implement the fact that decision-making and disease-spreading do not necessarily occur at the same time scales, we introduce the parameter $\alpha _1 \in {\mathbb {R}}$, which may be positive, negative, or zero. Large values of $|\alpha _1|$ correspond to frequent exposure to information regarding the disease. When $\alpha _1 > 0$, this corresponds to accurate information recommending disease avoidance, whereas when $\alpha _1<0$, this corresponds to (mis)-information which may suggest either the disease is harmless or that it is beneficial to contract the disease. The value $\alpha _1 = 0$ corresponds to no information regarding the disease, or equivalently, ignoring the disease’s existence. Consequently, the timescale of disease transmission is *t*, while the timescale at which individuals receive disease-related information is $|\alpha _1|^{-1} t$. There is also no reason that the timescale of disease transmission is equal to the timescale at which individuals either pay the cost of cooperating or reap the benefits of defecting in the presence of cooperators. To reflect this generality, we introduce the parameter $\alpha _2 > 0$, so that the timescale at which individuals receive PD payoffs is $\alpha _2^{-1} t$.

The decision whether to cooperate or defect is influenced by an individual’s social contacts, as described by a social network structure^34–37^. Recalling the PD payoffs which incorporate this network structure, (2), and with all of the above considerations in mind, the replicator equations for our hybrid SIS-PD model now read

4 $$\begin{aligned} \frac{dI}{dt}&=\left( [1-x(t)]\beta _D+x(t)\beta _C\right) I(t)(1-I(t))-\gamma I(t), \end{aligned}$$

5 $$\begin{aligned} \frac{dx}{dt}&=x(t)(1-x(t)) [\alpha _1(\beta _D-\beta _C)I(t)-\alpha _2 (C-N(k))]. \end{aligned}$$

Since $\beta _D > \beta _C$, if $\alpha _1 > 0$, and $C-N(k) >0$, the terms in the equation for the evolution of cooperators have opposite signs, resulting in a competition between avoidance of disease carriers and PD reward. We note that as soon as $C-N(k) \le 0$, the game ceases to be of PD-type. For further details concerning the derivation of these equations, see SI “Transmission rates for cooperators and defectors”.

Similar calculations lead to the replicator equations for the SIR-PD model

6 $$\begin{aligned} \dot{\mathscr {S}}(t)&= - \left( (1-x(t)) \beta _D + x(t) \beta _C \right) \mathscr {S}(t) I(t)\nonumber \\ \dot{I} (t)&= \left( (1-x(t)) \beta _D + x(t) \beta _C \right) \mathscr {S}(t) I(t) -\gamma I(t) \nonumber \\ \dot{\mathscr {R}}(t)&= \gamma I(t) \nonumber \\ \dot{x}(t)&= x(t)(1-x(t)) \left( \alpha _1(\beta _D - \beta _C) I(t) -\alpha _2(C-N(k))\right) \end{aligned}$$

Above, $\mathscr {S}$ is the number of susceptible individuals, and the quantities on the left are all differentiated with respect to time. If the portion $\gamma I(t)$ of infectious individuals recovers and acquires long-lasting immunity, we may describe the accumulated number of these individuals with a third compartment, namely $\mathscr {R}$, which is generally the number of recovered and immune, and/or deceased, individuals. The parameter $\gamma$ is the rate at which infected individuals either recover or die. If *D* is the average duration of the infection, irrespective of the outcome (recovery or death), then $\gamma = 1/D$, as in the SIS-PD model. This model is reasonably predictive for infectious diseases that are transmitted from human to human, and where recovery confers lasting resistance. Since $\dot{\mathscr {S}}+\dot{I}+\dot{\mathscr {R}} = 0$, the triplet $(x,I,\mathscr {S})$ describes the complete system; for further details see SI § “Transmission rates for cooperators and defectors”.

## Results

The network structure may cause the game to cease to be of PD type. For $k=1$, the game is always of PD type. For $k \ge 2$, the game is no longer of PD type if $C-N(k) \le 0$, which is equivalent to

7 $$\begin{aligned} \frac{R}{C} \ge k-1. \end{aligned}$$

If $\frac{R}{C}$ is large, corresponding to low costs of mitigation and/or high benefit of mutual cooperation, the game may cease to be of PD type for sufficiently small values of *k* such that the above inequality holds. However, since the right side of (7) tends to infinity when $k \rightarrow \infty,$ for sufficiently large values of *k*, the game is always of PD type. On the other hand, when $\frac{R}{C}$ is small, corresponding to either minimal benefit of mutual cooperation or extreme costs of mitigation, then there may be no value of *k* such that (7) holds, and so the game is always of PD type. Since our focus is the emergence of cooperation for a PD type game, we henceforth assume that the game is of PD type, and therefore we assume that $C-N(k) > 0$. We shall also assume that $\alpha _1 >0$, because if $\alpha _1 \le 0$, all individuals simply evolve to defect.

### SIR-PD equilibrium points

For the SIR-PD model, the complete set of equilibrium points consists of $(x, I, \mathscr {S}^*)$ with

$$\begin{aligned} x \in \{0, 1\}, \quad I=0, \quad 0 \le \mathscr {S}^* \le 1. \end{aligned}$$

The equilibrium points with $x=0$ are stable if $\beta _D \mathscr {S}^* \le \gamma$. When the reverse inequality holds, the equilibrium point is unstable. All equilibrium points with $x=1$ are unstable. For the details of these calculations, see SI “Calculation and classification of all equilibrium points in the SIR-PD model”.

### SIS-PD equilibrium points

The equilibrium points of the SIS-PD system are the set of (*x*, *I*):

$$\begin{aligned} \left\{ (0,0), (1, 0), \left( 0, 1 - \frac{\gamma }{\beta }_D \right) , \left( 1, 1-\frac{\gamma }{\beta }_C \right) , \left( x^*, I^*\right) \right\} , \quad x^* = \frac{\beta _D}{\beta _D - \beta _C} - \frac{\gamma }{(\beta _D - \beta _C)(1-I^*)}, \quad I^* = \frac{\alpha _2(C-N(k))}{\alpha _1(\beta _D - \beta _C)}. \end{aligned}$$

The equilibrium point, $(x^*, I^*)$, is well-defined as long as $x^* \in [0,1]$, and $I^* \in [0,1),$ since $C-N(k)>0$, and $\alpha _1, \alpha _2 > 0$. We compute that

$$\begin{aligned} I^*< 1 \iff \frac{\alpha _2 (C-N(k))}{\beta _D - \beta _C} < \alpha _1. \end{aligned}$$

We further compute

8 $$\begin{aligned} 0 \le x^*\le 1 \iff \check{\alpha }_1 \le \alpha _1\le \hat{\alpha }_1, \quad \check{\alpha }_1= \frac{\beta _D}{\beta _D - \gamma } \frac{\alpha _2 (C-N(k))}{\beta _D - \beta _C}\quad \mathrm{and} \quad \hat{\alpha }_1= \frac{\beta _C}{\beta _C - \gamma } \frac{\alpha _2 (C-N(k))}{\beta _D - \beta _C}. \end{aligned}$$

Since $1<\beta _D/(\beta _D-\gamma )$, this condition immediately implies $I^*<1$. We note that

$$\begin{aligned} \frac{\beta _D}{\beta _D - \gamma }< \frac{\beta _C}{\beta _C - \gamma } \implies \frac{\beta _D}{\beta _D - \gamma } \frac{\alpha _2 (C-N(k))}{\beta _D - \beta _C} < \frac{\beta _C}{\beta _C - \gamma } \frac{\alpha _2 (C-N(k))}{\beta _D - \beta _C}. \end{aligned}$$

Whenever it exists, the equilibrium point $(x^*, I^*)$ is *always *stable (and asymptotically stable).

**Table 1:** The asymptotically stable equilibrium points of the SIS-PD model in the specified ranges of $\alpha _1$, where $\check{\alpha }_1$ and $\hat{\alpha }_1$ are defined in (8).

Range	Equilibrium
$\alpha _1<\check{\alpha }_1$	$(0,1-\gamma /\beta _D)$
$\check{\alpha }_1\le \alpha _1\le \hat{\alpha }_1$	$(x^*,I^*)$
$\hat{\alpha }_1<\alpha _1$	$(1,1-\gamma /\beta _C)$

**Figure 2:**
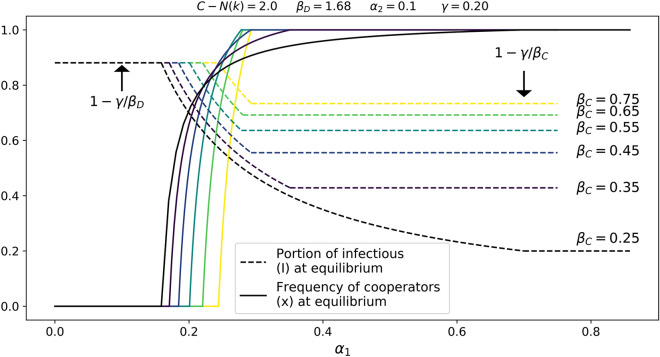
The values of $\beta _D$ and $\gamma$ above were suggested^54^; however these values can be modified to any disease parameters. Since it is the relationship between $\alpha _1$ and $\alpha _2$, rather than their individual values which affects the dynamics, we simply fix $\alpha _2=0.1$. The value of $\alpha _1$ ranges along the horizontal axis. The vertical axis is used to indicate both the frequency of cooperators, *x*, as well as the frequency of infectious individuals, *I*, within the population. For sufficiently large $\alpha _1$, the population evolves to cooperation. At the same time, the more effective the mitigation measures are, the lower $\beta _C$ is, which pushes the portion of infectious individuals to zero. More precisely, when $\alpha _1\ge \hat{\alpha }_1$, then $\lim _{\beta _C \searrow \gamma }I=0$.

**Figure 3:**
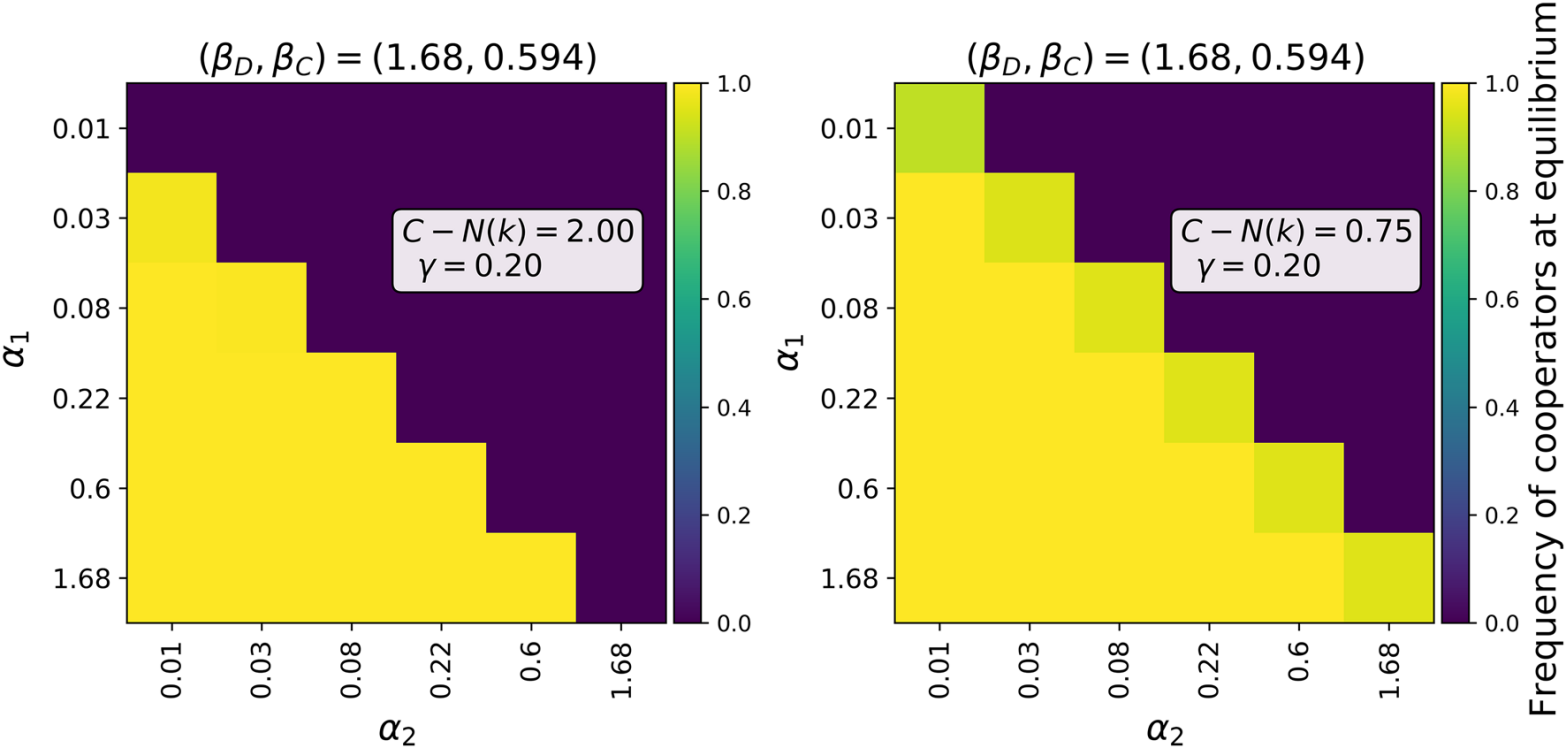
The evolution to cooperation depends on the relationship between $\alpha _1$ and $\alpha _2$, when the parameters $\beta _D$ and $\gamma$ are as suggested in^54^ and with $C-N(k)=2$ (left figure) or $C-N(k)=0.75$ (right figure). Here the value of $\beta _C$ corresponds to moderately effective mitigation measures. As $C-N(k)$ decreases, cooperating dominates a larger domain.

The equilibrium point (0, 0) is stable (and asymptotically stable) if

$$\begin{aligned} \beta _D < \gamma , \quad C-N(k)>0. \end{aligned}$$

Since $$x \beta _C + (1-x)\beta _D \le \beta _D$$,

$$\begin{aligned} \frac{dI}{dt} = \left[ \left( x \beta _C + (1-x)\beta _D \right) (1-I) -\gamma \right] I \le 0, \end{aligned}$$

with equality only if $I=0$, and hence there is no epidemic. The equilibrium point (1, 0) is never stable for PD payoffs. The equilibrium point $(0,1- \gamma /\beta _D)$, is well defined if $\beta _D\ge \gamma$, because $0 \le I \le 1$, and it is stable (and asymptotically stable) if

$$\begin{aligned} \alpha _1(\beta _D - \beta _C)(1-\gamma /\beta _D) < \alpha _2 (C-N(k)). \end{aligned}$$

For PD payoffs (2), this is equivalent to

9 $$\begin{aligned} \alpha _1< \check{\alpha }_1 \end{aligned}$$

The equilibrium point $(1,1-\gamma /\beta _C)$ is well defined if $\beta _C\ge \gamma$. It is stable (and asymptotically stable) if

$$\begin{aligned} \alpha _2 (C-N(k)) < \alpha _1(\beta _D - \beta _C) (1-\gamma /\beta _C). \end{aligned}$$

For PD payoffs (2), this equilibrium point is stable (and asymptotically stable) when

10 $$\begin{aligned} \hat{\alpha }_1 < \alpha _1. \end{aligned}$$

**Figure 4:**
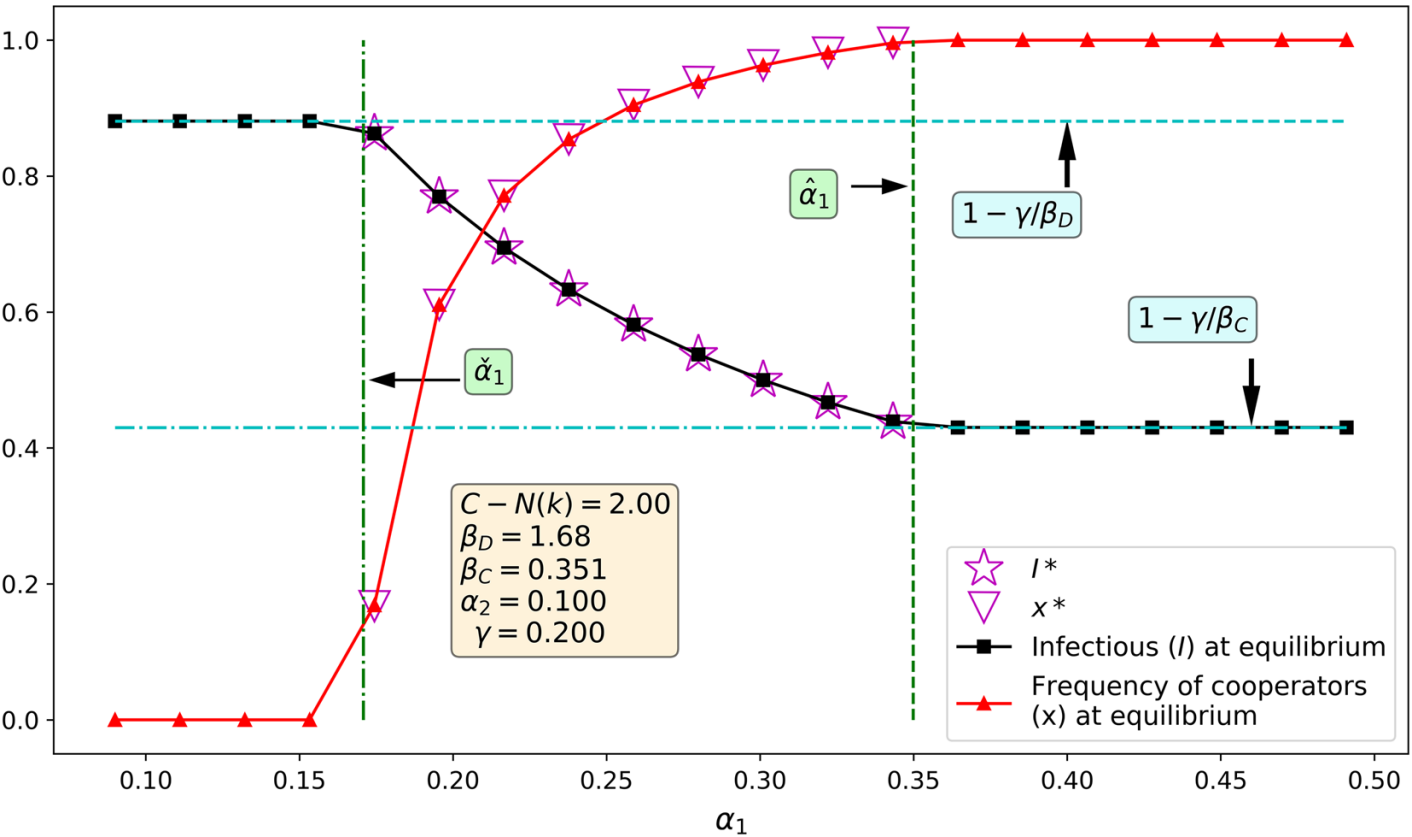
The results from numerical integration agree with the analytical results. The values of $\beta _D$ and $\gamma$ above were suggested^54^. The vertical axis is used to indicate both the frequency of cooperators, *x*, as well as the frequency of infectious individuals, *I*, within the population. Here the value of $\beta _C$ corresponds to mitigation measures which are more effective than in Fig. 3 but still imperfect.

Our results are summarised in Table 1. Figure 2 shows how the evolution of cooperation and the rate of infections depend on $\alpha _1$ and $\beta _C$ when $\beta _D=1.68$ and $\gamma =1/5$ as suggested in^54^. We note that these values were selected merely for the sake of visualisation, as our theoretical results hold for any parameter values. If both $\alpha _1$ and $\alpha _2$ vary, we obtain convergence to cooperation as shown in Fig. 3. Figure 4 shows that the numerical integration agrees perfectly with the analytical results. Note that the dependence on *both* $\alpha _1$ and $\alpha _2$ is actually a dependence on their *ratio*, since all the stability limits can be written as inequalities for the unknown $\alpha _1/\alpha _2$.

## Discussion

It has been suggested that mass media could be used to reduce HIV-infections^55^, and that this approach may explain the success in controlling HIV in Australia^56,57^. If an infectious disease, like HIV, does not confer immunity to those who recover from it, then SIS is a suitable model. The rate of spread for those who make no mitigation efforts, $\beta _D$, is strictly larger than the rate of spread for those who make mitigation efforts, $\beta _C$. Our results show that the relationship between the timescale of decision making and the timescale of PD payoffs is crucial. Decision-making is influenced by the speed at which individuals access or receive information upon which to base their decisions. It is reasonable to assume that the timescale of PD payoffs is similar to the timescale *t* for the spread of disease, or at least on the same order of magnitude. On the other hand, the speed at which individuals can access information could be much faster. This corresponds to $\alpha _1 \gg \alpha _2.$ When $\alpha _1>\hat{\alpha }_1$, the equilibrium point $(1, 1-\gamma /\beta _C)$ exists. Consequently, for sufficiently large $\alpha _1$, the *unique *equilibrium point of the system corresponds to total cooperation. Moreover, in this case the portion of the population which is infected tends to $1-\gamma /\beta _C$. We therefore also have

11 $$\begin{aligned} \lim _{\beta _C \searrow \gamma } 1-\frac{\gamma }{\beta _C} = 0. \end{aligned}$$

This shows that in the limit towards effective mitigation measures, the rate of the population which is infected tends to zero. We summarise these insights below.

In the context of a communicable disease which does not confer immunity, if accurate information is made available to all individuals quickly relative to the spread of the disease, all rational individuals acting in their best self interest will evolve to cooperate by taking measures to mitigate the spread. Simultaneously, increasingly effective mitigation measures drive the rate of infectious individuals to zero.

These insights suggest a strategy for controlling diseases which do not to confer immunity and may apply to SARS-CoV-2, as recent studies indicate that the disease might not confer immunity^58–60^. More generally, this strategy may be applied in the context of new diseases, for which it is unknown and unknowable whether contracting and recovering from the disease grants immunity^61^. Vaccines require time for development and testing^62^. It may therefore be prudent to use the SIS model for new communicable diseases. The value of $\alpha _1$ may be associated to the frequency of public service announcements (PSAs) which accurately convey effective mitigation measures. The more frequent the PSAs, the higher the value of $\alpha _1$. Our results prove that when $\alpha _1$ becomes sufficiently large, cooperation emerges, and the amount of infections can be controlled. Moreover, when mitigation measures are made increasingly effective, in the limit the frequency of infectious individuals tends to zero. The perceived benefit of defecting is defined by the PD payoffs (2), so that defecting is still perceived to offer benefits if others cooperate. The key to the evolution for cooperation is the time scale for decision making. This can be much faster than the time scale at which one can actually reap the benefits of defecting. When this is the case, the population evolves towards cooperation. Our results are not constrained to any specific disease, but rather suggest a general strategy to promote the evolution of cooperation in the Donor–Recipient game when linked to the SIS model. The SIS model has further applications to describing social and group dynamics^25^. Our model may thereby provide a mechanism for the evolution of cooperation in social contexts as well.

## Supplementary information

Supplementary information.
