# Supplementary material for: Decisions and disease: a mechanism for the evolution of cooperation
Source: Sci Rep. 2020 Aug 4;10:13113. doi: 10.1038/s41598-020-69546-2 (PMC7403384; doi:10.1038/s41598-020-69546-2)
Supplement: Supplementary file 1 — Supplementary information. [file 41598_2020_69546_MOESM1_ESM.pdf]

Supplementary information

Network reciprocity

The average number of social contacts is k, and is equal to the average node degree of the network. In order to incorporate network reciprocity and nonetheless retain the ability to obtain rigorous analytic results, we make a standard set of simplifying assumptions[35,37,38].

1. What two-step neighbors do is irrelevant. We consider only local frequencies of actor pairs: cooperators who have a cooperator in their immediate network, cooperators who have a defector in their immediate neighborhood, defectors who have a cooperator in their immediate neighborhood, and defectors who have a defector in their immediate neighborhood.
2. We assume weak selection, so that the dynamics of local frequencies decouples from the dynamics of global frequencies. Consequently the former reaches equilibrium before it affects the latter.
3. We assume the graph is degree-regular.

The result of these simplifying assumptions is that the payoffs T, and S shall be modified by the quantity

$$N(k) := \frac{(k+1)(R-P)-T+S}{(k+1)(k-2)}, \quad k \neq 2.$$

In our case, $P=0$, $S=-C$, and $T=R+C$, so we have

$$N(k) = \frac{Rk-2C}{(k+1)(k-2)}, \quad k \neq 2, \quad N(2) = R.$$

The derivation of $N(k)$ for $k \neq 2$ is based on[35,36], and for $k=2$, we refer to[37].

If $k=1$, the payoffs are $R > 0 = P$, $S = -\frac{R}{2}$, and $T = \frac{3R}{2}$, and consequently the game is of PD type since $T > R > P > S$. The game ceases to be of PD type when $N(k) - C \geq 0$, which is equivalent to

$$\frac{R}{C} \geq k-1. \tag{12}$$

Consequently, the benefit of mutual cooperation compared to the cost of mitigation determines whether the game is of PD type. For example, when $\frac{R}{C}$ is large, corresponding to low costs of mitigation and/or high benefit of mutual cooperation, the game may cease to be of PD type for values of sufficiently small values of k such that (12) holds. For sufficiently large values of k, the game remains of PD type. On the other hand, when $\frac{R}{C}$ is small, corresponding to either minimal benefit of mutual cooperation or extreme costs of mitigation, then there may be no value of k such that (12) holds, and so the game is always of PD type.

Calculation and classification of equilibrium points in the SIS-PD model.

Our equations in the SIS-PD model are:

$$\frac{dI}{dt} = ((1-x(t))\beta_D + x(t)\beta_C)I(t)(1-I(t)) - \gamma I(t) = f(I,x) \tag{13}$$

$$\frac{dx}{dt} = x(t)(1-x(t))[\alpha_1(\beta_D-\beta_C)I(t) - \alpha_2(C-N(k))] = g(I,x).$$

We compute that the equilibrium points of the system are the set of (x,I):

$$\left\{(0,0),(1,0),\left(0,1-\frac{\gamma}{\beta_D}\right),\left(1,1-\frac{\gamma}{\beta_C}\right),(x^*,I^*)\right\}$$

where

$$x^* = \frac{\beta_D}{\beta_D-\beta_C} - \frac{\gamma}{(\beta_D-\beta_C)(1-I^*)}, \quad I^* = \frac{\alpha_2(C-N(k))}{\alpha_1(\beta_D-\beta_C)}.$$

To determine the nature of the equilibrium points, that is whether they are (asymptotically) stable or unstable, we compute the Jacobian matrix

$$\begin{bmatrix} \frac{\partial f}{\partial I}, & \frac{\partial f}{\partial x} \\ \frac{\partial g}{\partial I}, & \frac{\partial g}{\partial x} \end{bmatrix}$$

whose entries are

$$\frac{\partial f}{\partial I} = (1-2I)((1-x)\beta_D + x\beta_C) - \gamma,$$

$$\frac{\partial f}{\partial x} = I(1-I)(\beta_C - \beta_D)$$

$$\frac{\partial g}{\partial I} = \alpha_1 x(1-x)(\beta_D - \beta_C),$$

$$\frac{\partial g}{\partial x} = (1-2x)\left[\alpha_1(\beta_D - \beta_C)I - \alpha_2(C - N(k))\right].$$

At the equilibrium point $(0,0)$ the Jacobian matrix is

$$\begin{bmatrix} \beta_D - \gamma & 0 \\ 0 & -\alpha_2(C - N(k)) \end{bmatrix}.$$

If the real parts of all eigenvalues are negative, then the equilibrium point is stable and asymptotically stable. This holds when

$$\beta_D < \gamma, \quad C - N(k) > 0.$$

The Jacobian matrix at the equilibrium point $(1,0)$ is

$$\begin{bmatrix} \beta_C - \gamma & 0 \\ 0 & \alpha_2(C - N(k)) \end{bmatrix}.$$

Since $\alpha_2 > 0$, and $C - N(k) > 0$, this equilibrium point is unstable.

The equilibrium point $(0, 1-\gamma/\beta_D)$ has Jacobian matrix

$$\begin{bmatrix} \gamma - \beta_D & (1-\gamma/\beta_D)(\gamma/\beta_D)(\beta_C - \beta_D) \\ 0 & \alpha_1(\beta_D - \beta_C)(1-\gamma/\beta_D) - \alpha_2(C - N(k)) \end{bmatrix}$$

Since $0 \leq I \leq 1$, this is well-defined if and only if

$$\gamma \leq \beta_D.$$

It is stable and asymptotically stable if

$$\alpha_1(\beta_D - \beta_C)(1-\gamma/\beta_D) < \alpha_2(C - N(k)).$$

For PD payoffs, and with $\alpha_1, \alpha_2 > 0$, this is equivalent to

$$\alpha_1 < \frac{\beta_D - \gamma}{\beta_D}\alpha_2\frac{C - N(k)}{\beta_D - \beta_C}.$$

The equilibrium point $(1, 1-\gamma/\beta_C)$ is well-defined if and only if

$$\gamma \leq \beta_C.$$

It has Jacobian matrix

$$\begin{bmatrix} \gamma - \beta_C & (1-\gamma/\beta_C)(\gamma/\beta_C)(\beta_C - \beta_D) \\ 0 & \alpha_2(C - N(k)) - \alpha_1(\beta_D - \beta_C)(1-\gamma/\beta_C) \end{bmatrix}$$

It is stable and asymptotically stable if

$$\alpha_2(C - N(k)) < \alpha_1(\beta_D - \beta_C)(1-\gamma/\beta_C).$$

The condition above is equivalent to

$$\frac{\beta_C}{\beta_C - \gamma}\frac{\alpha_2(C - N(k))}{\beta_D - \beta_C} < \alpha_1.$$

The equilibrium point, (x^*, I^*), exists as long as $x^* \in [0,1]$, and $I^* \in [0,1)$, since $C - N(k) > 0$, and $\alpha_1, \alpha_2 > 0$. We compute that

$$I^* < 1 \iff \frac{\alpha_2(C-N(k))}{\beta_D - \beta_C} < \alpha_1.$$

We further compute

$$0 \le x^* \iff \left(\frac{\beta_D}{\beta_D - \gamma}\right)\frac{\alpha_2(C-N(k))}{\beta_D - \beta_C} \le \alpha_1.$$

Since $1 < \frac{\beta_D}{\beta_D - \gamma}$, this condition immediately implies $I^* < 1$. We note that

$$\frac{\beta_D}{\beta_D - \gamma} < \frac{\beta_C}{\beta_C - \gamma}$$
$$\implies \left(\frac{\beta_D}{\beta_D - \gamma}\right)\frac{\alpha_2(C-N(k))}{\beta_D - \beta_C} < \frac{\beta_C}{\beta_C - \gamma}\frac{\alpha_2(C-N(k))}{\beta_D - \beta_C}.$$

We compute the Jacobian matrix

$$\begin{bmatrix} -\frac{I^*\gamma}{1-I^*} & I^*(1-I^*)(\beta_C - \beta_D) \\ \alpha_1 x^*(1-x^*)(\beta_D - \beta_C) & 0 \end{bmatrix},$$

Under these conditions, we compute that it is always stable (and asymptotically stable), because we compute that the eigenvalues of the Jacobian matrix have negative real part, since the matrix is of the form

$$\begin{bmatrix} - & - \\ + & 0 \end{bmatrix}.$$

Hence, the interesting values of α_1 are

$$\gamma \le \beta_D \text{ and } \alpha_1 < \left(\frac{\beta_D}{\beta_D - \gamma}\right)\frac{\alpha_2(C-N(k))}{\beta_D - \beta_C} \implies \exists \left(0, 1 - \frac{\gamma}{\beta_D}\right),$$

and this equilibrium point is stable and asymptotically stable. When α_1 is greater than or equal to this value,

$$\left(\frac{\beta_D}{\beta_D - \gamma}\right)\frac{\alpha_2(C-N(k))}{\beta_D - \beta_C} \le \alpha_1$$
$$\implies \exists (x^*, I^*) \text{ until } \alpha_1 = \frac{\beta_C}{\beta_C - \gamma}\frac{\alpha_2(C-N(k))}{\beta_D - \beta_C},$$

and this equilibrium point is stable and asymptotically stable. For $\alpha_1 > \frac{\beta_C}{\beta_C - \gamma}\frac{\alpha_2(C-N(k))}{\beta_D - \beta_C}$, $x^* > 1$, so this equilibrium point ceases to exist, but

$$\gamma \le \beta_C \text{ and } \frac{\beta_C}{\beta_C - \gamma}\frac{\alpha_2(C-N(k))}{\beta_D - \beta_C} < \alpha_1 \implies \exists \left(1, 1 - \frac{\gamma}{\beta_C}\right),$$

and this equilibrium point is stable and asymptotically stable.

Calculation and classification of all equilibrium points in the SIR-PD model

For the SIR-PD model, the equations are

$$\begin{aligned} \dot{\mathscr{S}} &= -((1-x)\beta_D + x\beta_C)\mathscr{S} I = \phi(x, I, \mathscr{S}) \\ \dot{I} &= -\dot{\mathscr{S}} - \gamma I = \psi(x, I, \mathscr{S}), \\ \dot{\mathscr{R}} &= \gamma I, \\ \dot{x}(t) &= x(1-x)\left[\alpha_1(\beta_D - \beta_C)I - \alpha_2(C - N(k))\right] = g(x, I). \end{aligned} \tag{14}$$

Since the equilibrium points require the left sides above to all vanish, this forces $I = 0$, since $\gamma \neq 0$. Consequently, it is straightforward to compute all of the equilibrium points. They are precisely the points $(x, I, \mathscr{S})$ listed below

$$\{(0,0,\mathscr{S}^*), (1,0,\mathscr{S}^*)\} \quad \mathscr{S}^* \in [0,1].$$

The Jacobian matrix is

$$\begin{bmatrix} \frac{\partial \phi}{\partial \mathscr{S}}, & \frac{\partial \phi}{\partial I}, & \frac{\partial \phi}{\partial x} \\ \frac{\partial \psi}{\partial \mathscr{S}}, & \frac{\partial \psi}{\partial I}, & \frac{\partial \psi}{\partial x} \\ \frac{\partial g}{\partial \mathscr{S}}, & \frac{\partial g}{\partial I}, & \frac{\partial g}{\partial x} \end{bmatrix},$$

and its entries are

$$\frac{\partial \phi}{\partial \mathscr{S}} = -\left((1-x)\beta_D + x\beta_C\right) I, \quad \frac{\partial \phi}{\partial I} = -\left((1-x)\beta_D + x\beta_C\right)\mathscr{S}, \quad \frac{\partial \phi}{\partial x} = (\beta_D - \beta_C)\mathscr{S} I$$
$$\frac{\partial \psi}{\partial \mathscr{S}} = \left((1-x)\beta_D + x\beta_C\right) I, \quad \frac{\partial \psi}{\partial \mathscr{S}} = \left((1-x)\beta_D + x\beta_C\right)\mathscr{S} - \gamma, \quad \frac{\partial \psi}{\partial x} = (\beta_C - \beta_D)\mathscr{S} I$$
$$\frac{\partial g}{\partial \mathscr{S}} = 0, \frac{\partial g}{\partial I} = x(1-x)\alpha_1(\beta_D - \beta_C), \quad \frac{\partial g}{\partial x} = (1-2x)\left[\alpha_1(\beta_D - \beta_C) I - \alpha_2(C - N(k))\right].$$

The equilibria with $x = 0$ have Jacobian matrix

$$\begin{bmatrix} 0 & -\beta_D \mathscr{S}^* & 0 \\ 0 & \beta_D \mathscr{S}^* - \gamma & 0 \\ 0 & 0 & -\alpha_2(C - N(k)) \end{bmatrix}$$

The equilibrium point is stable and asymptotically stable if $\beta_D \mathscr{S}^* < \gamma$. The equilibria with $x = 1$ have Jacobian matrix

$$\begin{bmatrix} 0 & -\beta_C \mathscr{S}^* & 0 \\ 0 & \beta_C \mathscr{S}^* - \gamma & 0 \\ 0 & 0 & \alpha_2(C - N(k)) \end{bmatrix}$$

Since $\alpha_2 > 0$, and $C - N(k) > 0$, this equilibrium is always unstable.

Transmission rates for cooperators and defectors

In the limit as $\alpha_2 \searrow 0$, that is, if the individual's perceived 'selfish gain or cost' as described by the PD payoffs vanishes, then the cooperators and defectors can be assigned separate rates of transmission. In other words, we can decompose the susceptible portion of the population into cooperators and defectors. We limit the discussion to the SIR model (14), as the idea is analogous for the SIS model. The rate at which individuals become susceptible according to (14) is,

$$\dot{\mathscr{S}} = -\beta \mathscr{S} I.$$

Let the portion of susceptible individuals consist of two parts:

$$\mathscr{S} = \mathscr{S}_C + \mathscr{S}_D,$$

where $\mathscr{S}_i$ the portion of infectious individuals that catch the disease at a rate β_i, for $i = C$ or $i = D$. Assuming $\beta_C < \beta_D$, we interpret this as C being individuals taking precautionary measures and thereby reducing their exposure to the disease, whereas D individuals do not change their behaviors. Let

$$x = \frac{\mathscr{S}_C}{\mathscr{S}_C + \mathscr{S}_D}. \tag{15}$$

Then if the transmission rates differ such that $\dot{\mathscr{S}}_C = -\beta_C \mathscr{S}_C I$ and $\dot{\mathscr{S}}_D = -\beta_D \mathscr{S}_D I$,

$$\dot{\mathscr{S}} = \dot{\mathscr{S}}_C + \dot{\mathscr{S}}_D = -\beta_C \mathscr{S}_C I - \beta_D \mathscr{S}_D I = -(\beta_C x + \beta_D(1-x))\mathscr{S} I \tag{16}$$

which motivates the term *effective transmission rate* for

$$\beta(t) = \beta_C x(t) + \beta_D(1 - x(t)).$$

Moreover

$$\dot{x} = \frac{\dot{\mathscr{S}}_C(\mathscr{S}_C+\mathscr{S}_D)-\mathscr{S}_C(\dot{\mathscr{S}}_C+\dot{\mathscr{S}}_D)}{(\mathscr{S}_C+\mathscr{S}_D)^2} = \frac{\dot{\mathscr{S}}_C\mathscr{S}_D-\mathscr{S}_C\dot{\mathscr{S}}_D}{(\mathscr{S}_C+\mathscr{S}_D)^2} = \frac{-\beta_C\mathscr{S}_C I\mathscr{S}_D+\mathscr{S}_C\beta_D\mathscr{S}_D I}{(\mathscr{S}_C+\mathscr{S}_D)^2} = \frac{\mathscr{S}_C}{\mathscr{S}_C+\mathscr{S}_D}\frac{(-\beta_C\mathscr{S}_D+\beta_D\mathscr{S}_D)I}{\mathscr{S}_C+\mathscr{S}_D}$$

$$= x\frac{\mathscr{S}_D}{\mathscr{S}_C+\mathscr{S}_D}(\beta_D-\beta_C)I = x\left(1-\frac{\mathscr{S}_C}{\mathscr{S}_C+\mathscr{S}_D}\right)(\beta_D-\beta_C)I = x(1-x)(\beta_D-\beta_C)I. \tag{17}$$

That is, in this limit as $\alpha_2 \searrow 0$, the portion x describes the fraction of cooperating and susceptible (healthy) individuals. This follows a dynamics reminiscent to evolutionary dynamics and presumes that the disease timescale and decision-making timescale are identical, corresponding to $\alpha_1 = 1$. However, cooperation in the sense we investigate here involves paying the PD-type payoff for any inconvenience associated to taking precautionary measures. Thus, we require $\alpha_2 > 0$. Consequently, the above interpretation of x, in (15), cannot be made. Instead, we keep the 'effective' transmission rate β as in (16) as a model for disease dynamics and modify the dynamics for x, so that there is a tendency to act selfishly due to the PD gains. This is similar to the approach of Poletti *et al.*[53]. They generalized the dynamics by working 'backwards,' in the sense that the effective transmission rate β in (16) and the dynamics of x are fixed by construction and not derived from (15). One can say that individuals *contribute* with β_D or β_C but *experience* the effect of both. Poletti *et al.* introduced payoffs that are dependent on the number of infectious individuals, and they combined those payoffs with the SIR model. They also observed that the timescales for disease dynamics and decision making need not be identical, and therefore it is reasonable to introduce parameters analogous to α_1 and α_2 as we do here.

Computer simulation details

All simulations were performed with the initial conditions $x(0) = 0.5$ and $I(0) = 0.001$ if otherwise is not stated. The choice of initial conditions does not change the convergence results, only the dynamics at small times. The ODEs are integrated using the Python routine `scipy.integrate.solve_ivp` with default settings. These simulations were used to produce Figure 4 which shows that the numerical integration agrees perfectly with the analytical results in the main text.
